# Molecular and cytological analyses reveal distinct transformations of intestinal epithelial cells during *Xenopus* metamorphosis

**DOI:** 10.1186/s13578-015-0065-3

**Published:** 2015-12-29

**Authors:** Morihiro Okada, Luan Wen, Thomas C. Miller, Dan Su, Yun-Bo Shi

**Affiliations:** Section on Molecular Morphogenesis, Program in Cellular Regulation and Metabolism (PCRM), Eunice Kennedy Shriver National Institute of Child Health and Human Development (NICHD), National Institutes of Health (NIH), 18 Library Dr., Bethesda, MD 20892 USA; Meso-Scale Discovery, Rockville, MD USA; Oncology Department, Zhejiang Provincial People’s Hospital, Hangzhou, China

**Keywords:** Thyroid hormone, Metamorphosis, *Xenopus laevis*, Thyroid hormone receptor, Stem cells, Intestine

## Abstract

**Background:**

The thyroid hormone (T3)-induced formation of adult intestine during amphibian metamorphosis resembles the maturation of the mammalian intestine during postembryonic development, the period around birth when plasma T3 level peaks. This process involves de novo formation of adult intestinal stem cells as well as the removal of the larval epithelial cells through apoptosis. Earlier studies have revealed a number of cytological and molecular markers for the epithelial cells undergoing different changes during metamorphosis. However, the lack of established double labeling has made it difficult to ascertain the identities of the metamorphosing epithelial cells.

**Results:**

Here, we carried out different double-staining with a number of cytological and molecular markers during T3-induced and natural metamorphosis in *Xenopus laevis.* Our studies demonstrated conclusively that the clusters of proliferating cells in the epithelium at the climax of metamorphosis are undifferentiated epithelial cells and express the well-known adult intestinal stem cell marker gene Lgr5. We further show that the adult stem cells and apoptotic larval epithelial cells are distinct epithelial cells during metamorphosis.

**Conclusions:**

Our findings suggest that morphologically identical larval epithelial cells choose two alternative paths: programmed cell death or dedifferentiation to form adult stem cells, in response to T3 during metamorphosis with apoptosis occurring prior to the formation of the proliferating adult stem cell clusters (islets).

## Background

Intestinal remodeling during *Xenopus* metamorphosis serves as an excellent model to study the development of vertebrate adult organ-specific adult stem cells, which are essential for physiological tissue renewal and regeneration. This transformation of the larval intestine to the adult form during amphibian metamorphosis involves the removal of larval epithelium and de novo formation of the adult epithelium with concurrent maturation of the other intestinal tissues in a process similar to the maturation of the mammalian intestine around birth [1–5]. The tadpole intestine consists of largely a monolayer of larval epithelial cells surrounded by thin layers of connective tissue and muscles. During metamorphosis, the larval epithelial cells undergo apoptosis and clusters of proliferating adult epithelial cells are formed de novo, which subsequently proliferate and differentiation to form a multiply folded adult epithelium surrounded by thick layers of connective tissue and muscles [1, 6–12]. Gene expression analyses of known adult stem cell markers of mammalian intestine, such as Lgr5 [13], suggest that the clusters of proliferating cells are adult stem cells.

Like all other processes during amphibian metamorphosis, intestinal remodeling is under the control of thyroid hormone (T3) [14, 15]. This process can be easily induced by adding physiological concentrations of T3 to premetamorphic tadpole rearing water or prevented by blocking the synthesis of endogenous T3. In addition, it is organ autonomous and can be induced with T3 even in intestinal organ cultures of premetamorphic tadpoles. Such properties makes intestinal remodeling a superior model to study the development of adult organ-specific stem cells as compared to the mammalian models, where it is difficult to manipulate the uterus-enclosed late stage embryos for such studies.

Earlier work in *Xenopus laevis* has shown that T3 induces the vast majority of the larval epithelial cells to undergo programmed cell death or apoptosis and that the proliferating adult epithelial cells are formed de novo, apparently from the dedifferentiation of a small number of larval epithelial cells, via a yet-unknown mechanism [1, 7–12, 16–19]. These proliferating adult epithelial cells can be easily identified as clusters of cells or islets that can be labeled with DNA synthesis markers, such as ^3^H and 5-bromo-2′-deoxyuridine, or strongly stained with methyl green-pyronin Y (MPGY) at the climax of metamorphosis [16–18, 20]. In addition, in situ hybridization analyses have shown that well-known markers of the adult mammalian intestinal stem cells, such as leucine-rich repeat-containing G-protein coupled receptor 5 (Lgr5) and Musashi-1 (Msi-1), are expressed in clusters of intestinal epithelial cells at the climax of metamorphosis, suggesting that the clusters or islets are proliferating adult stem cells. However, there has been no report of using double labeling to ascertain the identities and property of these cell clusters. Here by using a combination of different staining methods, we successfully carried out different double labeling that allowed us to conclusively demonstrate that the clusters of epithelial cells induced by T3 at the climax of intestinal metamorphosis are proliferating, Lgr5^+^ adult stem cells. We further show that these cells can be strongly stained with MPGY and lack intestinal fatty acid binding protein (IFABP), which is expressed in the differentiated epithelial cells. Finally, we demonstrated that apoptotic and the proliferating cells are distinct populations of epithelial cells at the climax of metamorphosis.

## Results and discussions

### Proliferating adult intestinal epithelial cells exist as cell clusters and lack the differentiation marker IFABP

**Fig. 1:**
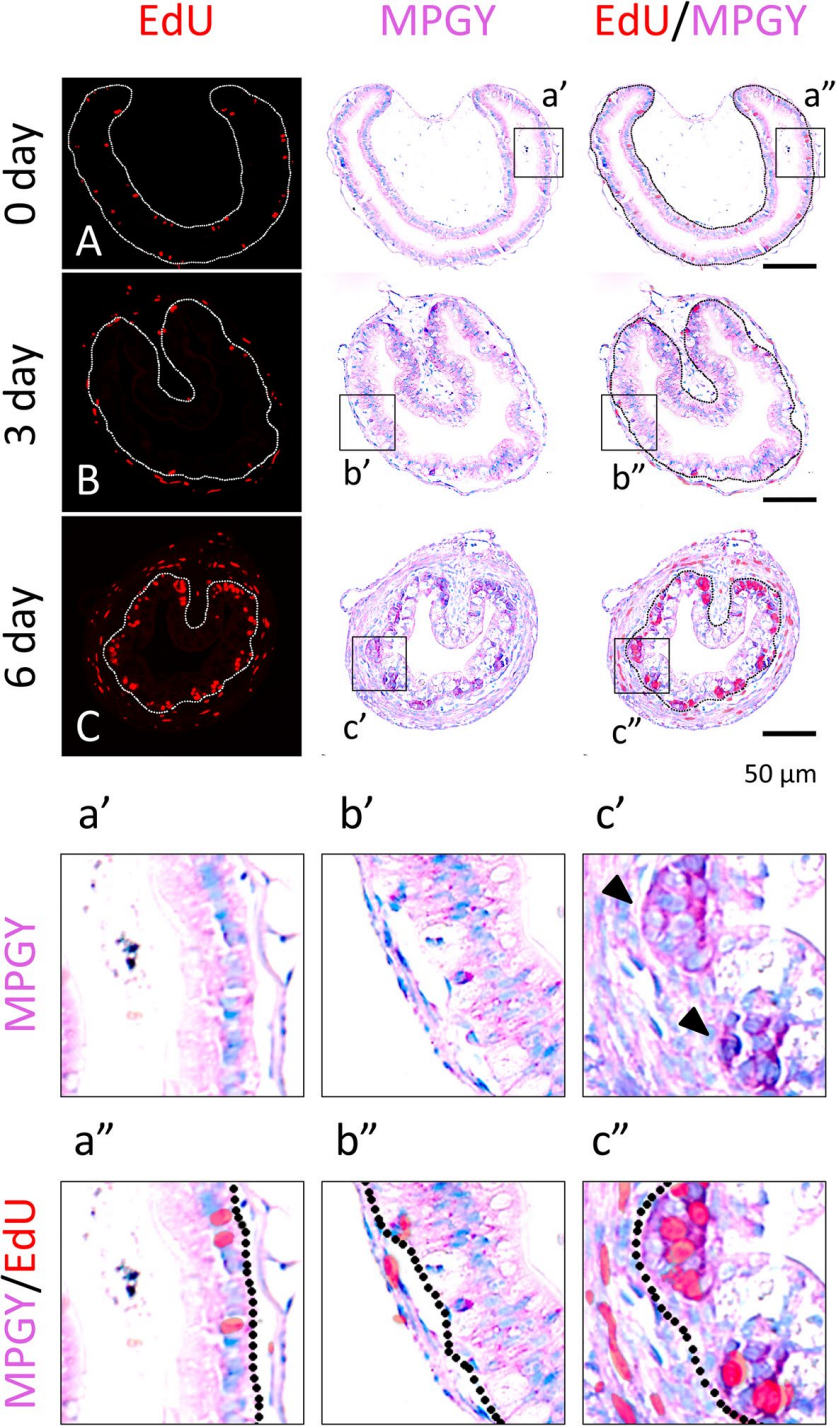
MGPY stains strongly the clusters (*islets*) of proliferating adult intestinal epithelial cells during T3-induced intestinal metamorphosis. Premetamorphic stage 54 tadpoles treated with 10 nM T3 for 0 (**A**), 3 (**B**), or 6 days (**C**) and were sacrificed 1 h after injection with EdU. Cross-sections of the intestine from the resulting tadpoles were double-stained for EdU and with MGPY. Higher magnifications of boxed areas in (**A**–**C**) are shown in (**a**′–**c**′) and (**a**″–**c**″). The approximate epithelium-mesenchyme boundary was drawn based on morphological differences between epithelial cells and mesenchyme cells in the pictures of the double-stained tissues, under enhanced contrast and/or brightness by using Photoshop, if needed (*dotted lines*). Note that the clusters (*islets*) of EdU labeled cells in the epithelium after 6 days of T3 treatment were strongly stained by MGPY (**C**, **c**″). *Arrowheads* indicate the clusters of proliferating cells or islets (**c**′)

The remodeling of the intestine leads to distinct changes in the morphology of the intestinal cross-section. This can be easily detected by staining the tissue sections with a mixture (MPGY) of methyl green, which stains DNA, and pyronin Y, which stains RNA [20–22]. Earlier studies have shown that MGPY stains strongly clusters of epithelial cells formed at the climax of metamorphosis or after T3 treatment, while the surrounding cells that are poor stained. As the epithelial cell clusters or islets at the climax of metamorphosis can be labeled with DNA synthesis markers [16, 20], it has been assumed that the clusters with active DNA synthesis are the same as those stained strongly with MGPY. To demonstrate this directly, we treated premetamorphic tadpoles at stage 54 with T3 for 0–6 days to induce metamorphosis. One hour prior to being sacrificed, the tadpoles were injected with 5-ethynyl-2′-deoxyuridine (EdU) to label the newly synthesized cellular DNA. The intestinal cross-sections from the resulting tadpoles were double-stained with MGPY and for EdU. As shown in Fig. 1, in premetamorphic intestine, the epithelium was uniformly stained with MGPY and some of the epithelial cells were EdU positive (Fig. 1A, a″). T3 treatment for 3 days had little effect on either intestinal morphology or staining (Fig. 1B, b″). However, after 6 days of T3 treatment, clusters of cells in the epithelium appeared and were more strongly stained by MGPY than the surrounding cells (Fig. 1C). Furthermore, these clusters were labeled by EdU (Fig. 1C, c″), indicating that the cell clusters strongly stained by MGPY are indeed the proliferating cell clusters.

**Fig. 2:**
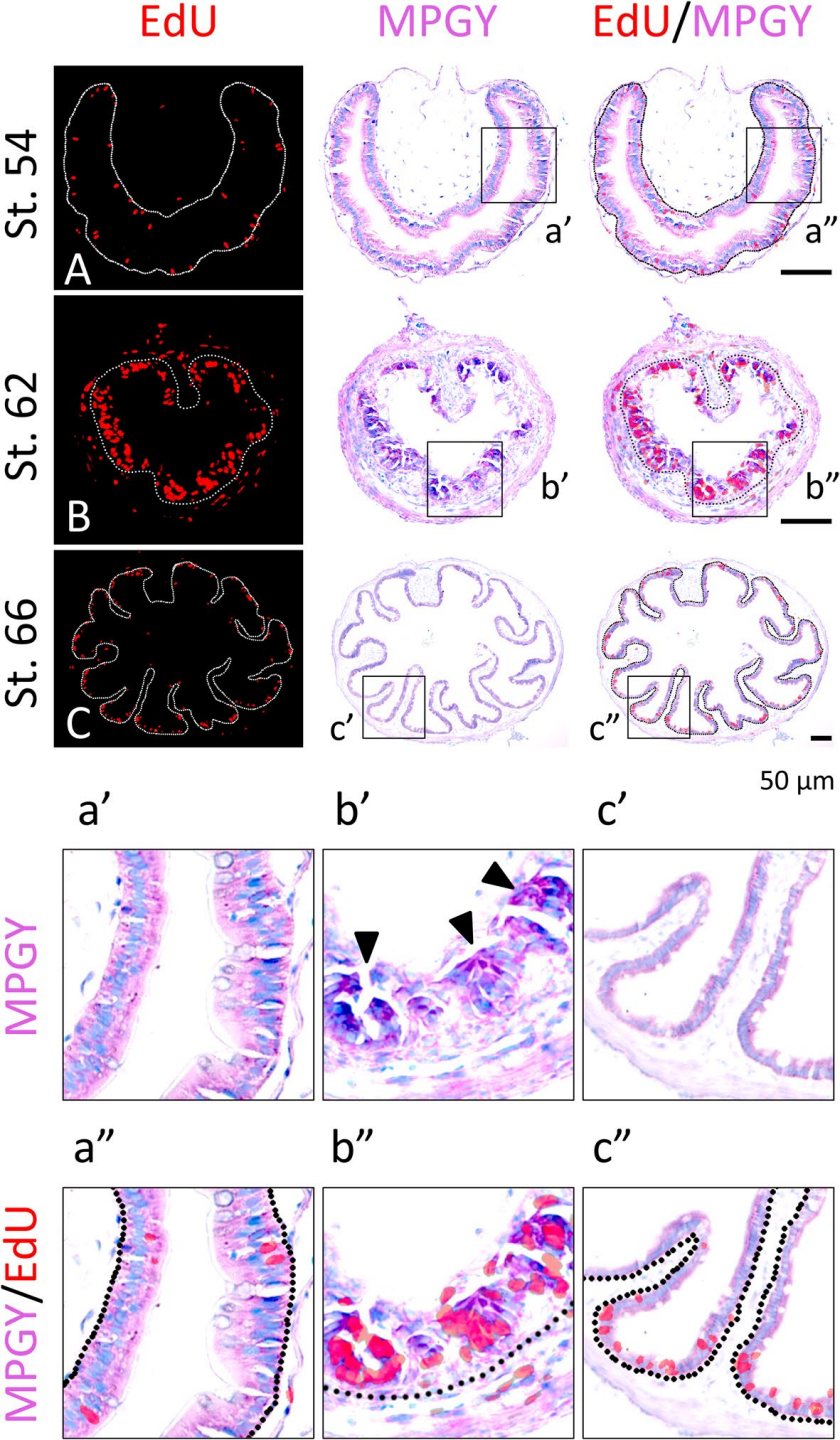
MGPY and EdU co-stain the clusters (*islets*) of proliferating adult intestinal epithelial cells at the climax of natural metamorphosis. Tadpoles at premetamorphic stage 54 (**A**), climax (**B** stage 62), and end of metamorphosis (**C** stage 66) were injected with EdU 1 h before being sacrificed. Cross-sections of the intestine from the resulting tadpoles were double-stained for EdU and with MGPY. Higher magnifications of boxed areas in (**A**–**C**) are shown in (**a**′–**c**′) and (**a**″–**c**″). The *dotted lines* depict the epithelium-mesenchyme boundary (see Fig. 1). *Arrowhead* indicates the clusters of proliferating cells or islets (**b**′)

To investigate this during natural metamorphosis, we carried out similar double labeling on intestinal cross-sections from tadpoles at premetamorphosis (stage 54), climax (stage 62), and end of metamorphosis (stage 66). The results showed that before or after metamorphosis, the epithelium were uniformly labeled with MGPY and some cells were also positive for EdU (Fig. 2A, C, a″, c″). Interestingly, the EdU label were preferentially at the bottom of the newly formed epithelial folds at the end of metamorphosis, suggesting that the proliferating adult cells become restricted to the bottom of the fold, which resembles the crypt in adult mammalian intestine where stem cells reside [23–25]. In contrast, at the climax of metamorphosis, clusters of epithelial cells were much more strongly stained by MGPY than the surrounding cells and these clusters were also labeled by EdU (Fig. 2B, b″), just like that during T3 induced metamorphosis. Thus, the newly formed epithelial cell clusters during metamorphosis are proliferating cells that are strongly stained by MGPY.

**Fig. 3:**
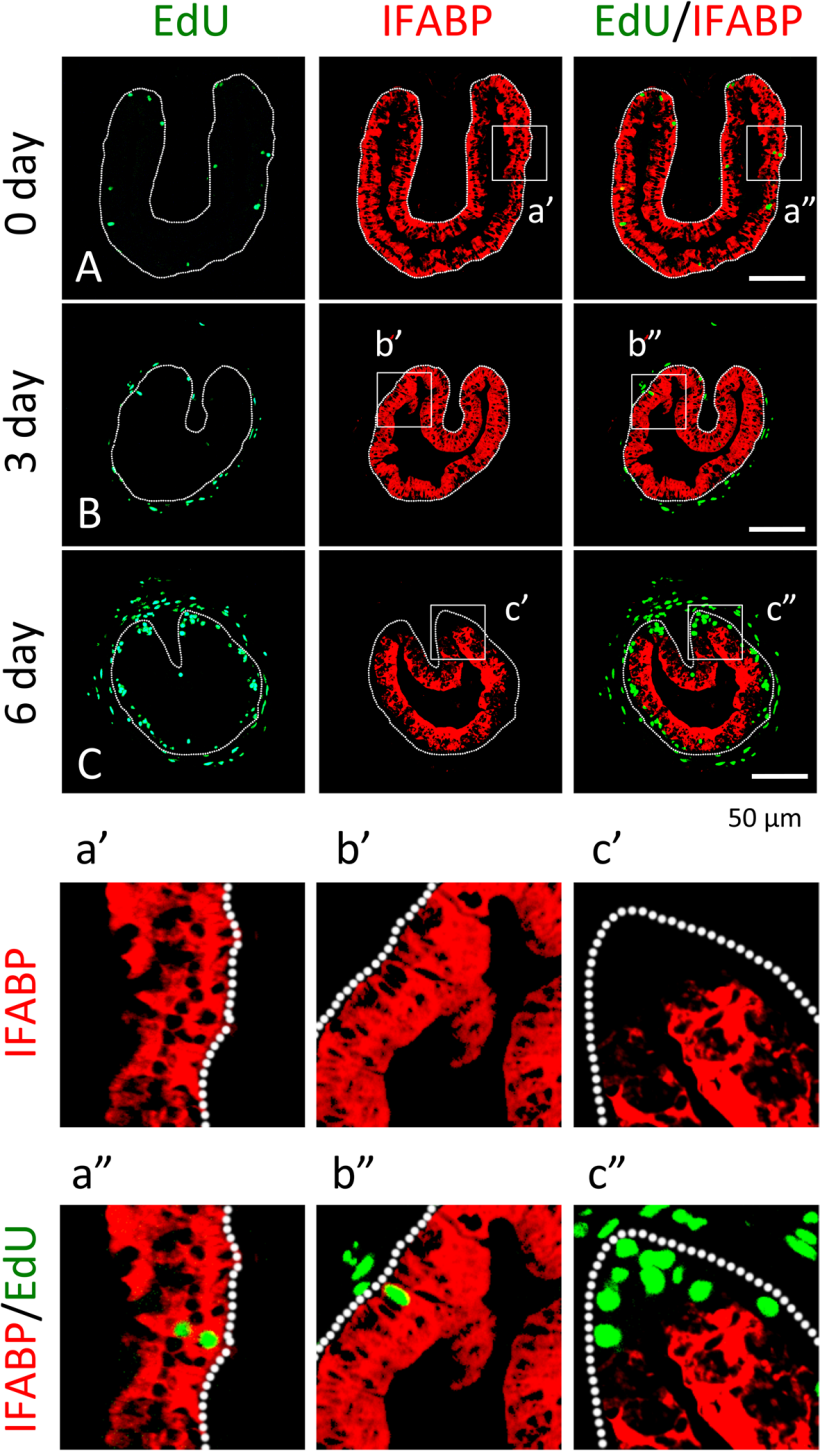
The newly formed proliferating adult intestinal epithelial cells during T3-induced metamorphosis have little or no expression of IFABP. Premetamorphic stage 54 tadpoles treated with 10 nM T3 for 0 (**A**), 3 (**B**), or 6 days (**C**) and were sacrificed 1 h after injection with EdU. Cross-sections of the intestine from the resulting tadpoles were double-stained for IFABP by immunohistochemistry and for EdU. Higher magnifications of boxed areas in (**A**–**C**) are shown in (**a**′–**c**′) and (**a**″–**c**″). The *dotted lines* depict the epithelium-mesenchyme boundary (see Fig. 1). Note that the EdU labeling revealed profound cell proliferation after T3 treatment. The proliferating cells in the epithelium after 6 days of T3 treatment were present mainly in clusters where IFABP staining was weak or absent (**C**, **c**″)

**Fig. 4:**
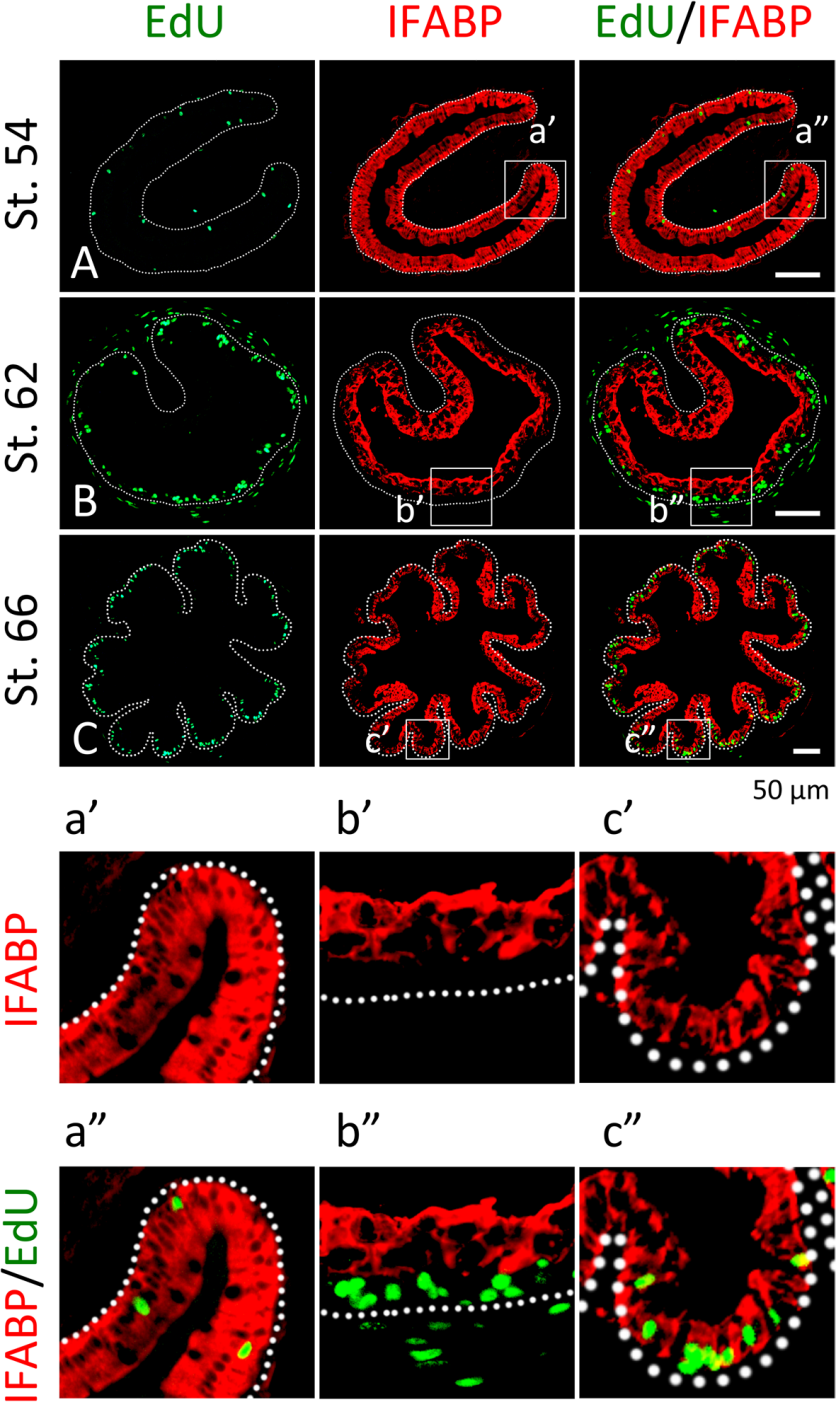
Clusters of proliferating adult intestinal epithelial cells at the climax of natural metamorphosis lack IFABP. Tadpoles at premetamorphic stage 54 (**A**), climax (**B** stage 62), and end of metamorphosis (**C**, stage 66) were injected with EdU 1 h before being sacrificed. Cross-sections of the intestine from the resulting tadpoles were double-stained for EdU and IFABP by immunohistochemistry. Higher magnifications of boxed areas in (**A**–**C**) are shown in (**a**′–**c**′) and (**a**″–**c**″). The *dotted lines* depict the epithelium-mesenchyme boundary (see Fig. 1). Note that the EdU-labeled proliferating cells in the epithelium were few and expressed IFABP at premetamorphosis (**A**) and increased in form of clustered cells that lacked IFABP at the climax of metamorphosis (**B**, **b**″). At the end of metamorphosis, EdU-labeled proliferating cells were localized mainly in the troughs of the epithelial folds where IFABP expression was low (**C**, **c**″)

Since differentiated larval epithelial cells are capable of proliferating, e.g., those at stage 54 (Figs. 1A, 2A), it is possible that the cells in the clusters formed at the climax of metamorphosis are differentiated cells. To investigate this, we carried out double-labeling of intestinal cross-sections during T3 (Fig. 3) or natural (Fig. 4) metamorphosis by using EdU for cell proliferation and immunohistochemistry for IFABP, a marker for differentiated intestinal epithelial cells [22, 26]. As shown in Fig. 3, after 0–3 days of T3 treatment, there were only low levels of EdU positive cells in the epithelium but the entire epithelium was uniformly labeled with anti-IFABP antibody (Fig. 3A, B). Furthermore, the EdU labeled epithelial cells were positive for IFABP (Fig. 3a″, b″). These findings are consistent with earlier observations showing that larval epithelial cells are mitotically active and express IFABP uniformly [16, 20, 22, 26]. After 6 days of T3 treatment, however, the number of EdU positive cells in the epithelium increased dramatically and existed as clusters in regions adjacent to the connective tissue and lacked IFABP signal (Fig. 3C, c″).

When we carried out similar analyses on intestinal cross-sections during natural metamorphosis, we also observed that at the climax of metamorphosis (stage 62), the EdU labeled cells were present as clusters between the luminal, larval epithelial cells that were positive for IFABP and the connective tissue (Fig. 4B, b″), while before (stage 54) or after (stage 66) metamorphosis (Fig. 4A, C, respectively), the EdU positive cells had IFABP, although at the troughs of the epithelial folds of post-metamorphic intestine (stage 66), the EdU positive cells had little or lower levels of IFABP (Fig. 4c″). These findings suggest that T3 induces the formation of clusters of proliferating cells that are dedifferentiated or undifferentiated. The same conclusion was reached when double-labeling was carried out with immunohistochemistry against IFABP and PCNA (proliferating cell nuclear antigen) (data not shown).

### The proliferating epithelial cell clusters express adult intestinal stem cell marker Lgr5

**Fig. 5:**
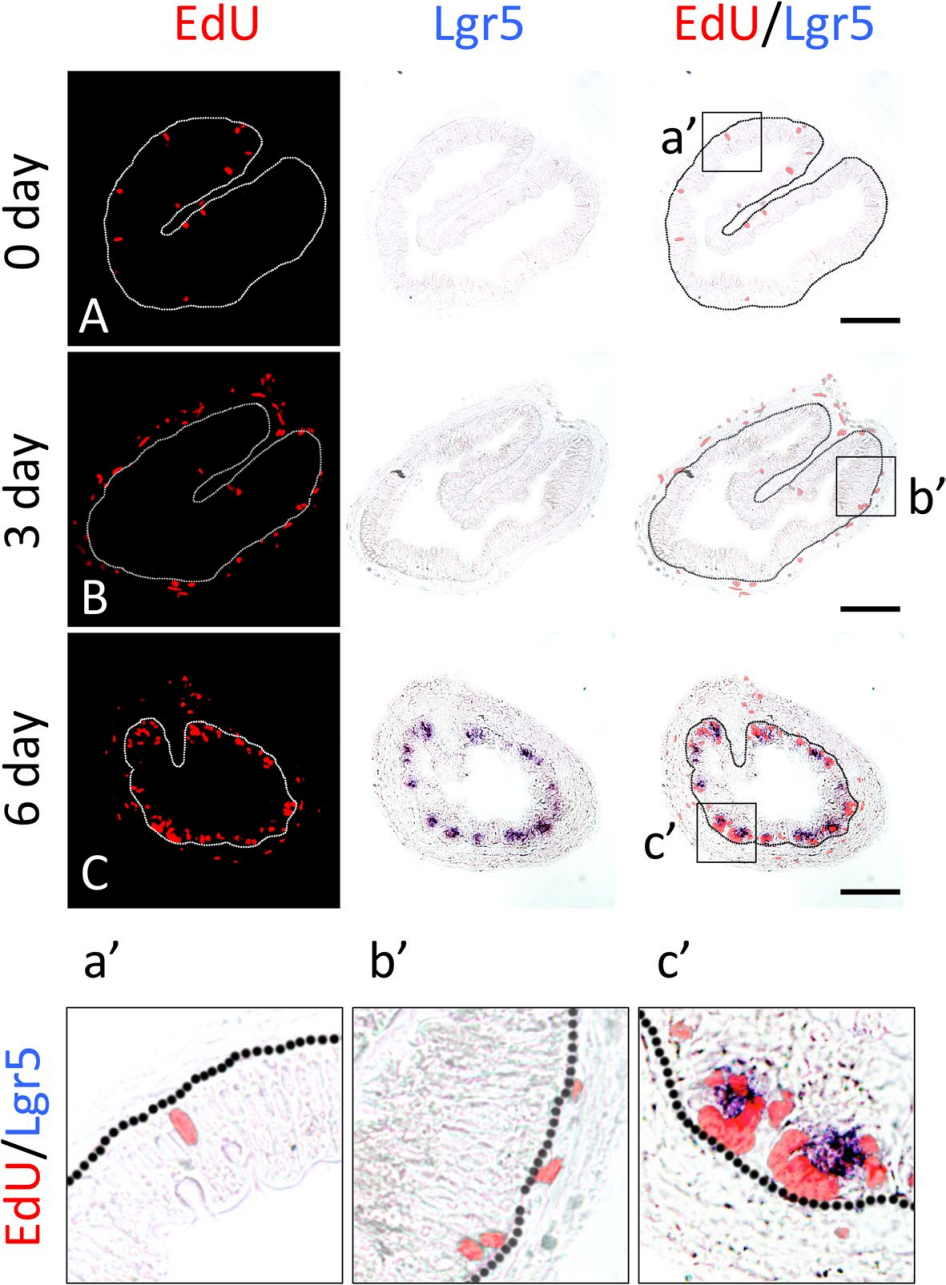
The EdU-labeled clusters (*islets*) of proliferating adult intestinal epithelial cells during T3-induced intestinal metamorphosis express the adult intestinal stem cell marker Lgr5. Premetamorphic stage 54 tadpoles treated with 10 nM T3 for 0 (**A**), 3 (**B**), or 6 days (**C**) and were sacrificed 1 h after injection with EdU. Cross-sections of the intestine from the resulting tadpoles were double-stained for Lgr5 by in situ hybridization and for EdU. Higher magnifications of *boxed* areas in (**A**–**C**) are shown in (**a**′–**c**′). The *dotted lines* depict the epithelium-mesenchyme boundary (see Fig. 1). Note that the clusters (*islets*) of EdU labeled cells in the epithelium after 6 days of T3 treatment had high levels of Lgr5 mRNA (**c**, **c**′)

**Fig. 6:**
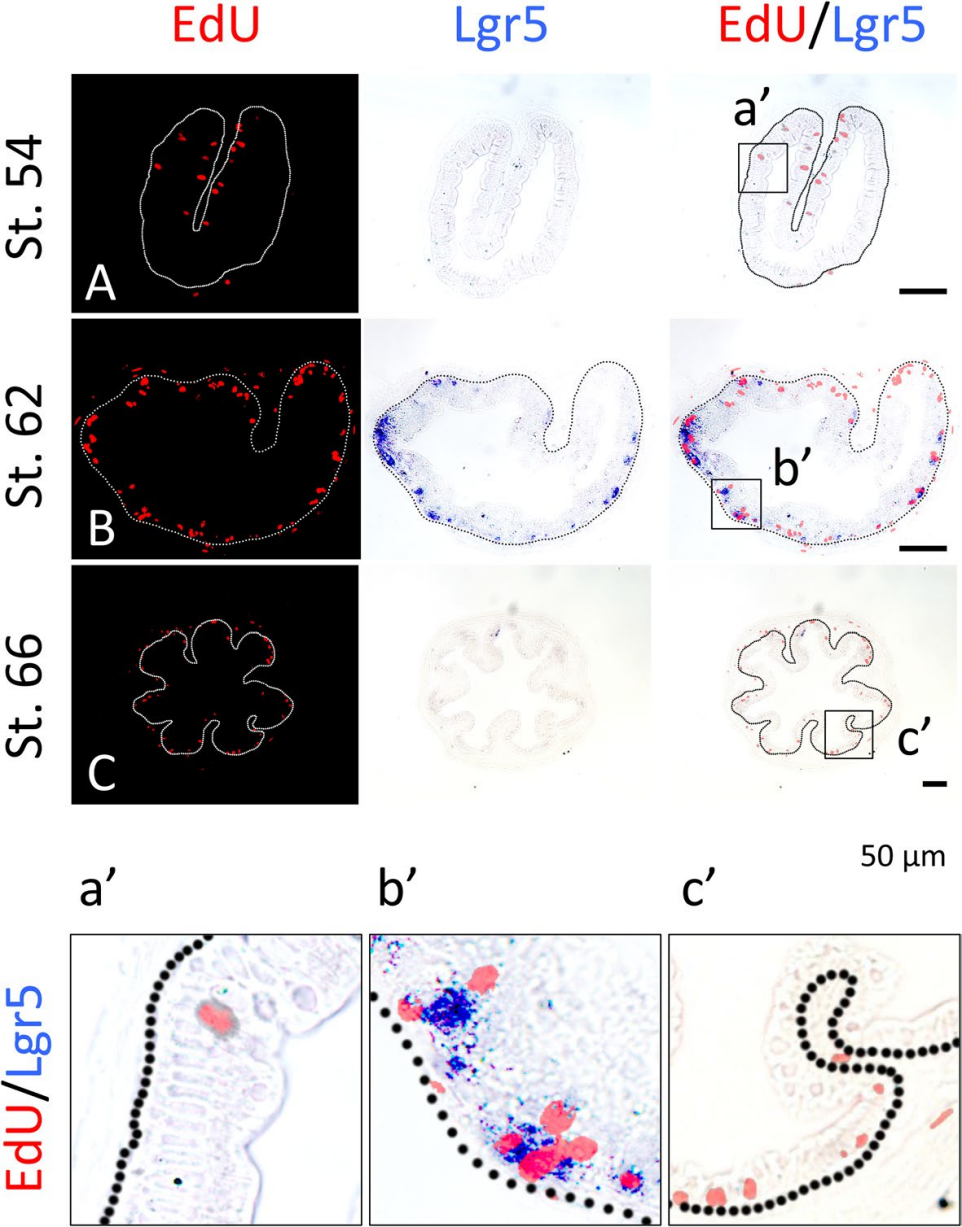
Lgr5 and EdU co-stain the clusters (*islets*) of proliferating adult intestinal epithelial cells at the climax of natural metamorphosis. Tadpoles at premetamorphic stage 54 (**A**), climax (**B** stage 62), and end of metamorphosis (**C** stage 66) were injected with EdU 1 h before being sacrificed. Cross-sections of the intestine from the resulting tadpoles were double-stained for EdU and Lgr5. Higher magnifications of boxed areas in (**A**–**C**) are shown in (**a**′–**c**′). The *dotted lines* depict the epithelium-mesenchyme boundary (see Fig. 1)

We have previously shown that the well-known adult mammalian intestinal stem cell marker Lgr5 is upregulated during metamorphosis and is expressed preferentially or specifically in the epithelial cell clusters formed during metamorphosis [13]. To determine if Lgr5^+^ clusters are expressed in the proliferating cell clusters of the epithelium, we carried out double-labeling with EdU and Lgr5 in situ hybridization for Lgr5 mRNA on intestinal cross-sections during natural or T3-induced metamorphosis. As shown in Fig. 5, after 0–3 day T3 treatment of premetamorphic tadpoles, there were few Lgr5^+^ cells and no cells were found to co-stain with Lgr5 and EdU (Fig. 5A, B, a′, b′). However, after 6 days of treatment, Lgr5 positive cell clusters were numerous in the epithelium and these clusters were labeled with EdU (Fig. 5C, c′). Similarly, we found that at the climax (stage 62) during natural metamorphosis, the epithelial cell clusters were co-labeled with Lgr5 and EdU (Fig. 6b, b′). However, no such clusters were present before (stage 54) (Fig. 6A) or after (stage 66) (Fig. 6C) metamorphosis. Thus, the proliferating epithelial cells in the clusters formed during metamorphosis are adult stem cells.

### Apoptotic and proliferating cells represent distinct populations of epithelial cells at the climax of metamorphosis

**Fig. 7:**
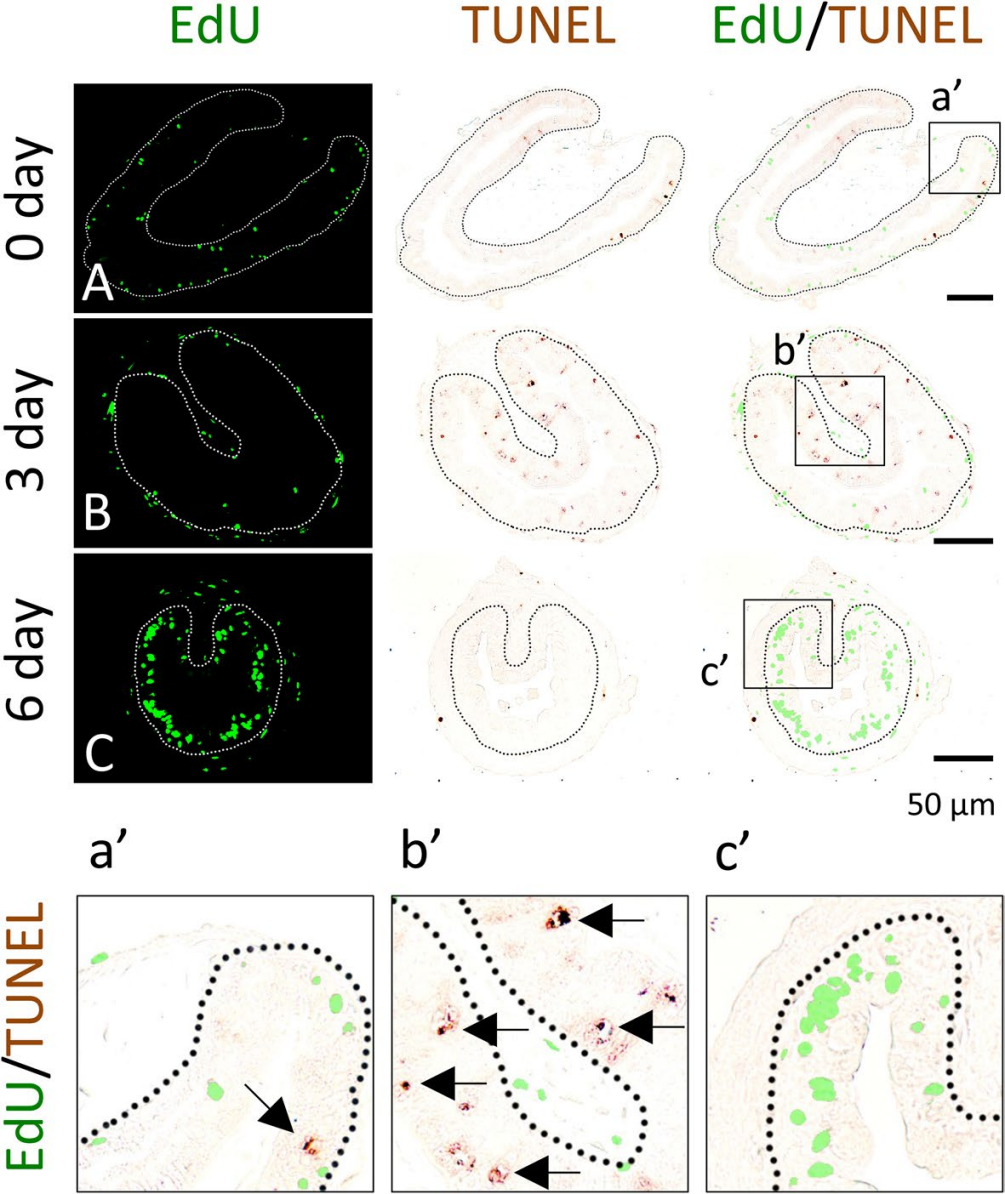
EdU and TUNEL-labeling reveals that apoptotic and proliferating cells are non-overlapping epithelial cells during T3-induced intestinal metamorphosis. Premetamorphic stage 54 tadpoles treated with 10 nM T3 for 0 (**A**), 3 (**B**), or 6 days (**C**) and were sacrificed 1 h after injection with EdU. Cross-sections of the intestine from the resulting tadpoles were double-stained for apoptosis by TUNEL and for EdU. Higher magnifications of boxed areas in (**A**–**C**) are shown in (**a**′–**c**′). The *dotted lines* depict the epithelium-mesenchyme boundary (see Fig. 1). Note that apoptosis in the epithelium occurred prior to the appearance of the clusters (*islets*) of EdU labeled cells and in distinct epithelial cells during T3 treatment (**C**, **c**′)

**Fig. 8:**
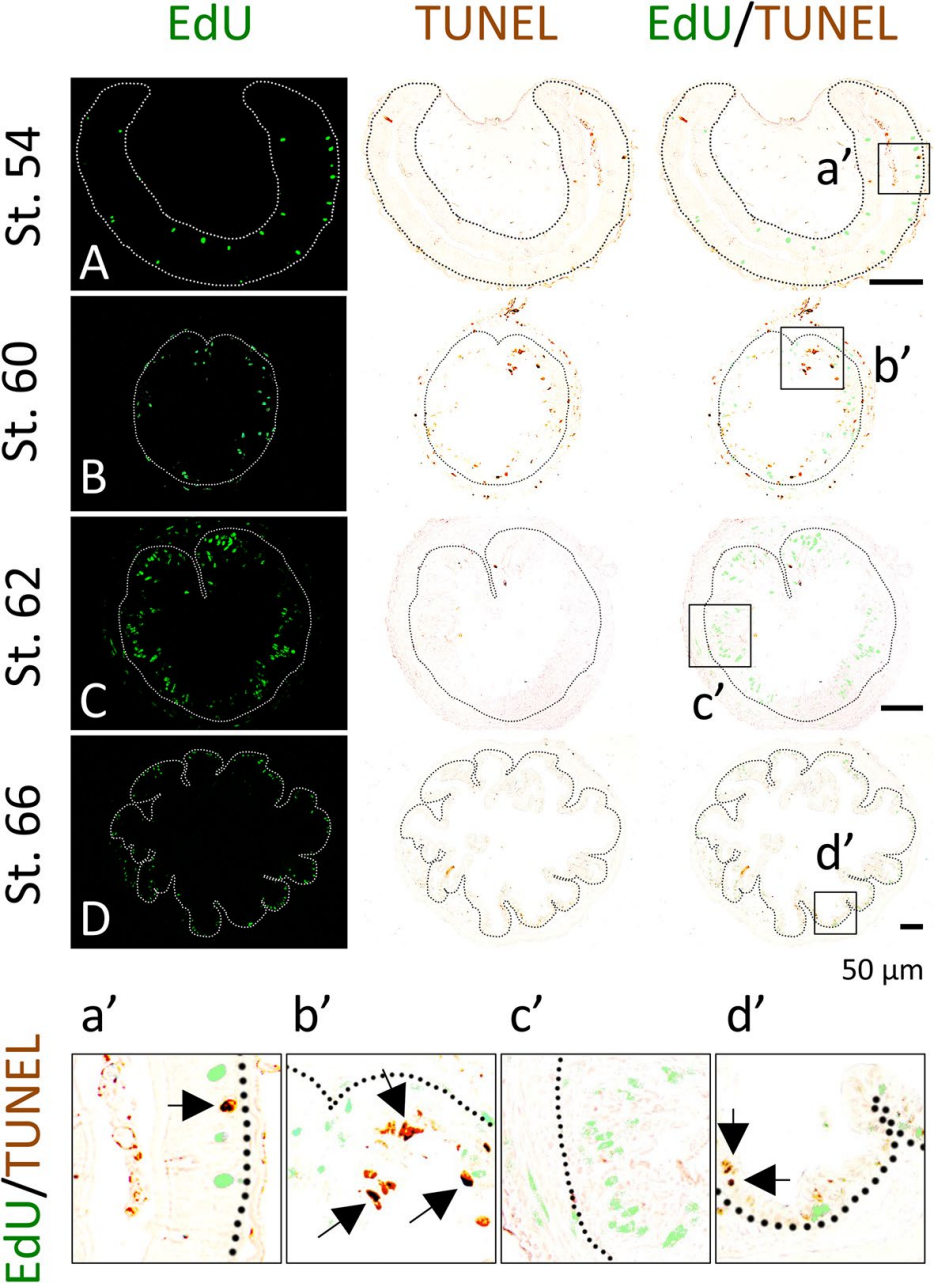
Epithelial apoptosis takes place prior to the appearance of clusters (*islets*) of proliferating adult intestinal stem cells during natural metamorphosis. Tadpoles at premetamorphic stage 54 (**A**), climax (**B** stage 60, **C** stage 62), and end of metamorphosis (**D** stage 66) were injected with EdU 1 h before being sacrificed. Cross-sections of the intestine from the resulting tadpoles were double-stained for apoptosis by TUNEL and for EdU. Higher magnifications of boxed areas in (**A**–**D**) are shown in (**a**′–**d**′). The *dotted lines* depict the epithelium-mesenchyme boundary (see Fig. 1)

T3 induces both larval epithelial cell death and adult epithelial development. We next used double-labeling to simultaneously detect apoptotic cells with TUNEL and proliferating cells with EdU. Consistent with earlier reports [27], after 3 days of T3 treatment of premetamorphic tadpoles, larval epithelial cell death could be detected by TUNEL (Fig. 7B) and few apoptotic cells were detected after 6 days, although many EdU positive cells were present (Fig. 7C). No co-staining of any cells by TUNEL and EdU was detected throughout the T3 treatment. Similarly, during natural metamorphosis, there were many TUNEL and EdU positive cells at stage 60 (climax of metamorphosis) but no co-stained cells were detected (Fig. 8B, b′). Like what was observed during T3 treatment, apoptotic labeling peaked before cell proliferation, with little apoptotic signal detected by stage 62 when EdU labeling was the strongest (Fig. 8C, c′). These findings indicate that T3-induced apoptosis occurs earlier than the massive proliferation of the adult stem cells during metamorphosis and that the proliferating cells and apoptotic cells are distinct cell populations in the intestinal epithelium at the climax of metamorphosis.

## Conclusion

Adult organ-specific stem cells are critical for organ homeostasis, repair, and regeneration and mis-regulation of such stem cells often leads to diseases such as cancer. Thus, extensive studies have been carried out to understand the regulation of organ-specific stem cells as well as cancer stem cells [28–36]. Intestinal remodeling during amphibian metamorphosis resembles the maturation of mammalian intestine around birth and thus has served as a model to study the development of adult organ-specific stem cells in vertebrates [2, 4, 5, 9, 37–43]. The advancements in genetic approaches for gene function studies in vivo, such as the knockout and knockin in *Xenopus* [44–46], undoubtedly further enhance the value of this unique model system for studying adult organ-specific stem cells. While earlier single labeling studies have provided valuable information for analyzing cell transformations in the epithelium, the lack of double-labeling has hindered analyses and/or interpretations regarding adult stem cells. Here, we have adapted different protocols that allowed us to double label different epithelial cells with several combinations of different labeling methods, including chemical labeling with EdU, staining with MGPY, in situ hybridization, and immunohistochemistry. These have allowed us to directly demonstrate experimentally for the first time that adult intestinal stem cells formed during metamorphosis are the proliferating cell clusters formed at the climax of metamorphosis. Considering our earlier findings that the adult stem cells are derived from larval epithelium [12, 19], our double-labeling studies of proliferating and apoptotic cells indicate that in response to T3, the epithelial cells take two mutually exclusive pathways, apoptosis or dedifferentiation followed by proliferation, which leads to the formation of the adult intestinal epithelium. Finally, our findings here pave the way to use any one of the labeling methods in this study to analyze stem cell development during metamorphosis.

## Methods

### Animals and treatments

Wild-type *X. laevis* tadpoles were reared in the laboratory or purchased from Nasco or Xenopus 1. The tadpoles were staged based on Nieuwkoop and Faber [47]. Premetamorphic *X. laevis* tadpoles at stage 54 were treated with 10 nM T3 for 0–6 days at 18 °C. At least 3 tadpoles were analyzed for each stage or day of T3 treatment. All animal studies were done in accordance with the guidelines established by the National Institute of Child Health and Human Development Animal Use and Care Committee.

### In situ hybridization

The in situ probe for Lgr5 was made as described previously [13]. Intestinal fragments were isolated from the anterior part of the small intestine of tadpoles at indicated stages, fixed in 4 % MOPS/EGTA/magnesium sulfate/formaldehyde buffer (MEMFA), followed by cryosectioning. Tissue sections cut at 7 µm were subjected to in situ hybridization by using the antisense probe as previously described [48]. For double staining with EdU staining, the sections were first processed for digoxygenin in situ hybridization, and then the slides were washed in 1× phosphate buffered saline plus 0.05 % Tween-20 for 5 min, followed by EdU staining.

### 5-Ethynyl-2′-deoxyuridine (EdU) labeling

EdU staining was performed as described [49]. Briefly, 6.7, 40 and 40 µL of 2.5-mg/mL EdU were injected into stage 54, 62, and 66 tadpoles, respectively. One hour after injection, the tadpoles were sacrificed, and the intestine was fixed in 4 % MEMFA and processed for cryosectioning. EdU was detected by using the Click-iT Plus EdU Alexa Fluor 594 Imaging kit (Life Technologies) according to supplier’s instructions.

### Immunohistochemistry

To identify differentiated intestinal absorptive cells, the sections were incubated with the rabbit anti-IFABP (intestinal fatty acid binding protein) antibody (diluted 1:500; [22]) overnight at 4 °C. Samples were washed several times with 1× phosphate buffered saline and primary antibodies were detected by using Alexa Fluor 568 Goat Anti-Rabbit IgG (H+L) Antibody (diluted 1:100; molecular probes). For double labeling with EdU staining, the sections were first processed for immunostaining, and then the slides were washed in 1× phosphate buffered saline plus 0.05 % Tween-20 for 5 min, followed by EdU staining.

### Methyl green-pyronin Y (MPGY) staining

Sections were stained with MPGY (Muto), a mixture of methyl green, which binds strongly to DNA, and pyronin Y, which binds strongly to RNA, for 5 min at room temperature [22]. Adult epithelial stem/progenitor cells were intensely stained red because of their RNA-rich cytoplasm [3]. For double staining with EdU labeling, the sections were first processed for EdU staining. After photographing the EdU labeling, the slides were washed in 1X Phosphate Buffered Saline plus 0.05 % Tween-20 for 5 min, followed by MPGY staining. The image of the MPGY staining was taken. The images from MPGY and EdU staining from the same slide were merged by using Adobe Photoshop CS5.1 to determine whether MPGY and EdU labeled the same cells.

### TUNEL assays

TUNEL (terminal deoxyribonucleotidyl transferase-mediated dUTP-biotin nick end labeling) assays were performed by using DeadEnd™ Colorimetric TUNEL System (Promega) as described [50]. For double staining, EdU staining was performed after the TUNEL assays.

## Abbreviations

T3: Thyroid hormone
TR: Thyroid hormone receptor
IFABP: Intestinal fatty acid binding protein
TUNEL: Terminal deoxyribonucleotidyl transferase-mediated dUTP-biotin nick end labeling
MPGY: Methyl green pyronin Y
EdU: 5-Ethynyl-2′-deoxyuridine
